# Dual Targeting of Y-Box Binding Protein-1 and Akt Inhibits Proliferation and Enhances the Chemosensitivity of Colorectal Cancer Cells

**DOI:** 10.3390/cancers11040562

**Published:** 2019-04-19

**Authors:** Eva Maier, Felix Attenberger, Aadhya Tiwari, Konstanze Lettau, Simone Rebholz, Birgit Fehrenbacher, Martin Schaller, Cihan Gani, Mahmoud Toulany

**Affiliations:** 1 Division of Radiobiology & Molecular Environmental Research, Department of Radiation Oncology, University of Tuebingen, 72076 Tuebingen, Germany; eva_maier@gmx.de (E.M.); felix.attenberger@web.de (F.A.); aadhya.tiwari@uni-tuebingen.de (A.T.); konstanze.lettau@student.uni-tuebingen.de (K.L.); simone.rebholz@klinikum.uni-tuebingen.de (S.R.); 2 German Cancer Consortium (DKTK), Partner Site Tuebingen, and German Research Center (DKFZ), 69120 Heidelberg, Germany; Cihan.Gani@med.uni-tuebingen.de; 3 Department of Dermatology, University of Tuebingen, 72076 Tuebingen, Germany; Birgit.Fehrenbacher@med.uni-tuebingen.de (B.F.); Martin.Schaller@med.uni-tuebingen.de (M.S.); 4 Department of Radiation Oncology, University of Tuebingen, 72076 Tuebingen, Germany

**Keywords:** Y-box binding protein 1, colorectal cancer, 5-fluorouracil, RSK, Akt

## Abstract

KRAS-mutated colorectal cancers (CRCs) are resistant to cetuximab treatment. The multifunctional Y-box binding protein 1 (YB-1) is overexpressed in CRC and is associated with chemoresistance. In this study, the effects of oncogenic mutated KRAS(G12V) and KRAS(G13D) on YB-1 phosphorylation were investigated in CRC cells. The effects of the inhibition of p90 ribosomal S6 kinase (RSK) on YB-1 phosphorylation, cell proliferation and survival were tested with and without treatment with 5-fluorouracil using pharmacological inhibitors and siRNA. YB-1 phosphorylation status and subcellular distribution in CRC patient tissues were determined by immunofluorescence staining and confocal microscopy. Endogenous expression of mutated KRAS(G13D) and conditional expression of KRAS(G12V) significantly stimulated YB-1 phosphorylation via RSK and were associated with cetuximab resistance. Inhibition of YB-1 by targeting RSK stimulated the Akt signaling pathway, and this stimulation occurred independently of KRAS mutational status. Akt activation interfered with the antiproliferative effect of the RSK inhibitor. Consequently, dual targeting of RSK and Akt efficiently inhibited cell proliferation in KRAS(G13D)-mutated HCT116 and KRAS wild-type SW48 cells. Treatment with 5-fluorouracil (5-FU) significantly enhanced YB-1 phosphorylation in KRAS(G13D)-mutated HCT116 cells but not in KRAS wild-type SW48 cells. Dual targeting of Akt and RSK sensitized HCT116 cells to 5-FU by stimulating 5-FU-induced apoptosis and inhibiting repair of 5-FU-induced DNA damage. YB-1 was highly phosphorylated in CRC patient tumor tissues and was mainly localized in the nucleus. Together, dual targeting of RSK and Akt may be an alternative molecular targeting approach to cetuximab for treating CRC in which YB-1 is highly phosphorylated.

## 1. Introduction

Colorectal cancer (CRC) is the second most commonly diagnosed cancer in women and the third most common cancer in men, and CRC is one of the leading causes of cancer death [1]. The first-line treatment for advanced CRC is 5-fluorouracil (5-FU)-based chemotherapy in combination with other cytotoxic drugs, such as leucovorin and oxaliplatin. Another approach for treating CRC is therapy with monoclonal antibodies, such as cetuximab, which target the epidermal growth factor receptor (EGFR) [2,3]. Administration of cetuximab is limited to patients with KRAS wild-type tumors because KRAS mutations are predictive markers for resistance to EGFR-targeted therapy [4,5,6]. Thus, approximately 40% of all CRC patients who harbor KRAS mutations [7] are not eligible for treatment with cetuximab. Additionally, 50–65% of KRAS wild-type patients are resistant to EGFR-targeted therapy [8,9]. Likewise, response rates of only 30–60% for 5-FU-based drug regimens have been reported [10,11]. This lack of therapy response in advanced CRC patients indicates the need for additional investigations for effective targeted therapy approaches to overcome resistance to conventional therapies.

The multifunctional Y-box binding protein 1 (YB-1) is a member of the cold shock protein superfamily containing a highly conserved nucleic acid-binding motif called the cold shock domain (CSD) [12]. The CSD enables YB-1 to interact with both DNA and RNA [13], acting as a transcription and translation factor [14]. YB-1 has been reported to be an important oncoprotein affecting many hallmarks of cancer, and it plays an important role in tumor cell proliferation and progression as well as in protecting cancer cells from apoptosis [12]. Furthermore, YB-1 has been described to interact with damaged DNA and DNA repair proteins, and it is involved in DNA repair [15,16,17]. YB-1 is overexpressed in a variety of cancers [14], including colorectal cancer [18,19]. A high expression of YB-1 in CRC patients is associated with poor clinical outcome [18,19,20]. YB-1 functions as a transcription factor for oncogenes, such as EGFR and Her2. YB-1 promotes tumorigenesis in different tumor entities, e.g., in spinal chordoma through activation of the EGFR/Akt pathway [21] and in melanoma by influencing the epithelial-to-mesenchymal transition [22]. Thus, YB-1 expression represents a negative prognostic factor in cancer patients with different tumor entities [22,23,24].

YB-1 expression is associated with resistance to chemotherapy agents, including cisplatin and gemcitabine [25,26]. YB-1 has been reported to be involved in CRC resistance to 5-FU [27]. To gain its function in affecting cell proliferation and survival, YB-1 must be present in the nucleus and activated via phosphorylation at serine 102 (S102) by p90 ribosomal S6 kinase (RSK) [28,29]. Our recent study described that following cellular stress, such as oncogenic KRAS expression, EGF treatment and ionizing radiation exposure, RSK translocates to the nucleus and phosphorylates nuclear YB-1 in breast cancer cells, but YB-1 does not translocate to the nucleus in response to these stimuli [29]. As a direct substrate of RSK, YB-1 is regulated via the mitogen-activated protein kinase (MAPK) pathway. Thus, YB-1 downstream of KRAS is constitutively active, as reported in KRAS-mutated breast cancer cells [30]. Small molecule RSK inhibitors, such as LJI308, have been described to prevent YB-1 S102 phosphorylation [31,32].

The present study investigated the activation status of YB-1 in cetuximab-sensitive KRAS wild-type and cetuximab-resistant KRAS-mutated CRC cells. The antiproliferative and chemosensitizing effects of the RSK inhibitor, LJI308, were investigated in CRC cells with different KRAS mutational statuses. The data indicated that the expression of mutated KRAS stimulates YB-1 phosphorylation. Due to the reactivation of Akt after RSK inhibition, dual targeting of RSK and Akt, but not single targeting of RSK, is an effective approach to block cell proliferation and induce chemosensitization in CRC cells.

## 2. Results

### 2.1. Mutation in KRAS Gene Diminishes the Antiproliferative Effect of Cetuximab in CRC Cells 

**Figure 1:**
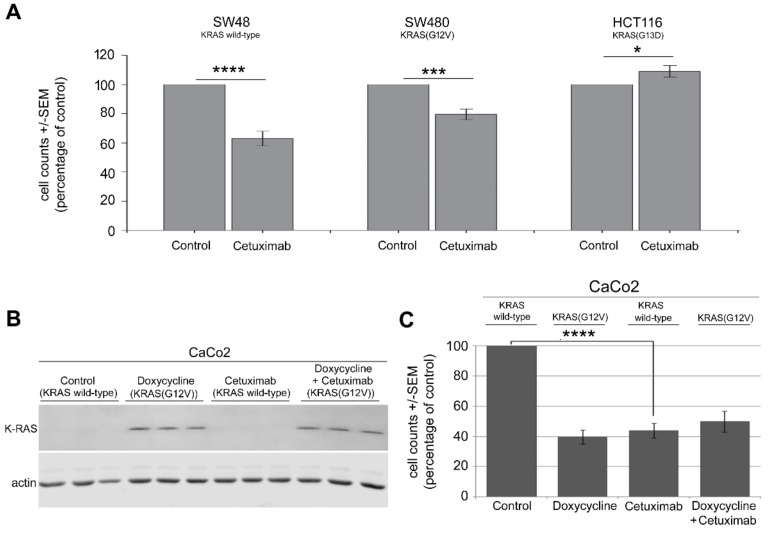
Mutation in KRAS gene diminishes the antiproliferative effect of cetuximab in colorectal cancer (CRC) cells. (**A**) KRAS wild-type SW48, KRAS(G12V)-mutated SW480 and KRAS(G13D)-mutated HCT116 cells, (**B**,**C**) KRAS wild-type CaCo2 cells with doxycycline-inducible KRAS(G12V) were treated with cetuximab (100 ng/mL) for 72 h, and a proliferation assay was performed. Asterisks indicate a statistically significant antiproliferative effect of cetuximab normalized to 1 in nontreated controls (* *p* ≤ 0.05, *** *p* ≤ 0.0001 and **** *p* ≤ 0.00001; 9 data points from three biologically independent experiments in SW48 and HCT116 cells; and 11 data points from two biologically independent experiments in SW480 cells). Western blot data show the expression of KRAS(G12V) 24 h after treatment with doxycycline. Actin was detected as a loading control.

Cetuximab, an anti-EGFR antibody, does not improve the treatment outcome of CRC patients harboring KRAS mutations [4,5,6]. Thus, cetuximab treatment is limited to patients with wild-type KRAS. The present study investigated the effect of cetuximab on the proliferation of CRC cells expressing wild-type KRAS and two distinct KRAS mutations in codon 12 (G12V) and codon 13 (G13D). A proliferation assay was used to examine the sensitivity of KRAS wild-type SW48 cells, KRAS(G12V)-mutated SW480 cells and KRAS(G13D)-mutated HCT116 cells to cetuximab. Cetuximab treatment (100 ng/ml for 3 days) strongly inhibited cell proliferation in KRAS wild-type SW48 cells but only moderately affected KRAS(G12V)-mutated SW480 cells (Figure 1A). Cetuximab did not reduce the proliferation of KRAS(G13D)-mutated HCT116 cells (Figure 1A). The effect of the KRAS(G12V) mutation on cetuximab response was also tested in an isogenic cell system. Briefly, CaCo2 cells were stably transfected with a doxycycline-inducible KRAS-mutated plasmid [33], which allowed for conditional expression of mutated KRAS(G12V) (Figure 1B). To test the effect of KRAS(G12V), cells were treated with doxycycline (2 µg/mL) 24 h prior to treatment with cetuximab, which led to the expression of KRAS(G12V) (Figure 1B) in association with reduced cell proliferation (Figure 1C). Cetuximab inhibited cell proliferation in parental cells but not in KRAS(G12V)-mutated cells (Figure 1C). To rule out the cytotoxic or cytostatic effect of doxycycline in reduced proliferation of CaCo2 cells, KRAS-mutated HCT116 and KRAS wild-type SW48 cells were treated with doxycycline (2 µg/mL), and the proliferation assay was performed after 72 h. As shown in Figure S1, doxycycline treatment did not affect proliferation of either cell line tested (Figure S1).

### 2.2. 5-FU Induces YB-1 Phosphorylation at S102 in KRAS(G13D)-Mutated HCT116 Cells but Not in KRAS Wild-Type SW48 Cells

**Figure 2:**
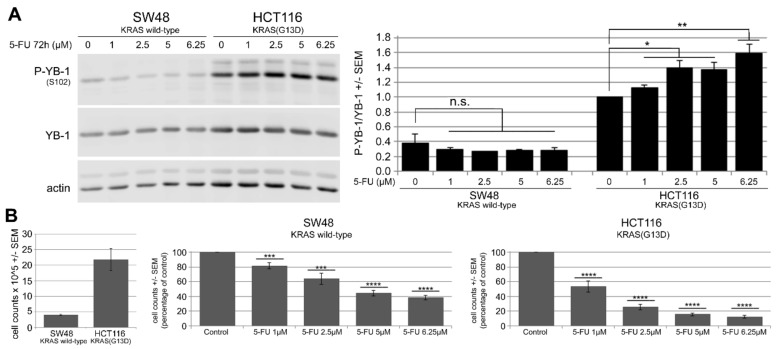
5-FU induces Y-box binding protein 1 (YB-1) phosphorylation at S102 in KRAS(G13D)-mutated HCT116 cells but not in KRAS wild-type SW48 cells. (**A**) KRAS wild-type SW48 and KRAS(G13D)-mutated HCT116 cells were treated with increasing concentrations of 5-FU for 72 h. Thereafter, protein samples were isolated, and phosphorylation of YB-1 was analyzed by Western blotting using a phospho-specific antibody. Actin was detected as the loading control. The histogram represents the mean ratio of phosphorylated YB-1 (P-YB-1)/YB-1 from three independent experiments normalized to untreated HCT116 control cells. (**B**) A proliferation assay was performed following the same treatment conditions. Histograms indicate the mean number of cells after treatment with the indicated concentrations of 5-FU normalized to the control condition in each cell line (9 data points from three biologically independent experiments). Asterisks indicate a significant antiproliferative effect of 5-FU as analyzed by Student’s *t*-test (* *p* ≤ 0.05, ** *p* ≤ 0.01, *** *p* ≤ 0.001, and **** *p* ≤ 0.0001; n.s.: nonsignificant). (**B**, left part) Comparison of absolute cell counts of control conditions in SW48 and HCT116 cells.

YB-1 is overexpressed in many CRC cells, and high expression of YB-1 is correlated with a lower overall and disease-free survival [18,19]. Because YB-1 activation has been described to be involved in chemoresponse, the pattern of YB-1 phosphorylation in cetuximab-sensitive SW48 cells and cetuximab-resistant HCT116 was investigated after treatment with 5-FU. Western blot analysis, including densitometry values (Figure 2A), indicated that 5-FU significantly induced YB-1 phosphorylation at S102 in cetuximab-resistant KRAS(G13D)-mutated HCT116 cells in a dose-dependent manner. However, phosphorylation of YB-1 in cetuximab-sensitive SW48 cells was slightly reduced, which was not significant (Figure 2A). KRAS-mutated cells proliferated more than KRAS wild-type cells. 5-FU inhibited cell proliferation in both cell lines in a dose-dependent manner (Figure 2B). However, the effect was stronger in HCT116 cells compared to that in SW48 cells.

### 2.3. Targeting RSK by LJI308 Inhibits Phosphorylation of YB-1 at S102 in CRC Cells

**Figure 3:**
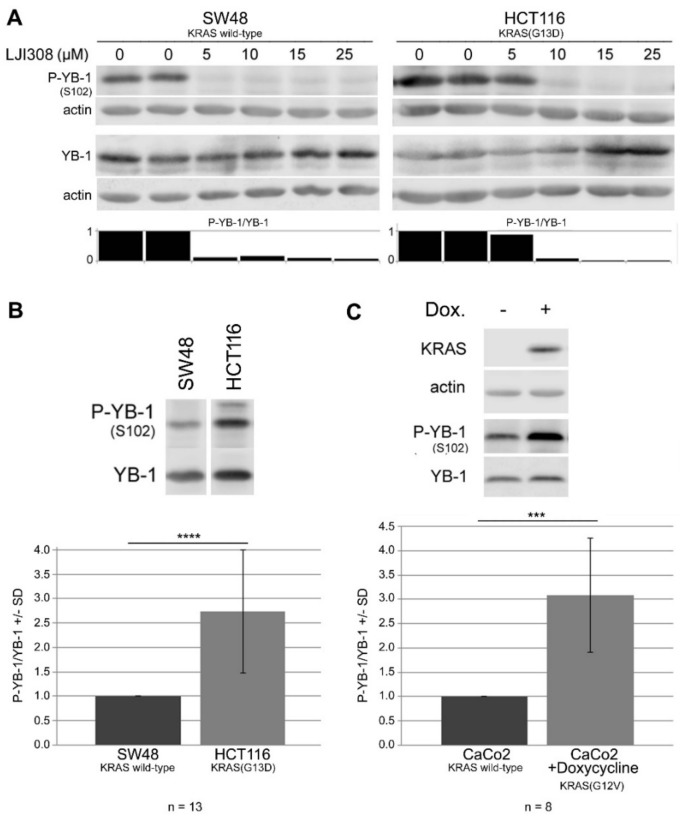
Targeting p90 ribosomal S6 kinase (RSK) by LJI308 inhibits phosphorylation of YB-1 at S102 in CRC cells. (**A**) KRAS wild-type SW48 and KRAS(G13D)-mutated HCT116 cells were treated with increasing concentrations of the RSK inhibitor, LJI308, for 20 h. The level of YB-1 phosphorylation was analyzed by Western blotting. (**B**) The mean ratio of phospho-YB-1 to YB-1 was evaluated by densitometry under unstimulated basal conditions in the indicated number of experiments in KRAS wild-type SW48 and KRAS(G13D)-mutated HCT116 cells (**C**) as well as in CaCo2 cells with and without conditional expression of the KRAS(G12V) mutation in the indicated number of experiments. (**B**,**C**) Representative Western blots are shown. Asterisks indicate a statistically significant difference in the phosphorylation of YB-1 between the indicated cells/conditions (*** *p* ≤ 0.001 and **** *p* ≤ 0.0001).

The phosphoinositide 3-kinase/Akt (PI3K/Akt) and mitogen-activated protein kinase/extracellular signal-regulated kinase (MAPK/ERK) pathways are responsible for the activation of YB-1 by phosphorylation at S102 [28,34]. Furthermore, the phosphorylation of YB-1 at S102 in breast cancer cells is mainly mediated through the MAPK pathway via the p90 ribosomal S6 kinase [28]. Therefore, the present study investigated if RSK targeting is a suitable approach to inhibit YB-1 phosphorylation and interfere with the proliferation of CRC cells by using the small molecule RSK inhibitor, LJI308, which inhibits the activation of all four RSK isoforms [31]. A dose-response experiment showed that LJI308 completely inhibited phosphorylated YB-1 (P-YB-1) in SW48 cells at a concentration of 5 µM. A similar level of inhibition was achieved in HCT116 cells by LJI308 at a concentration of 10 µM (Figure 3A). Because HCT116 cells harbor a mutation in KRAS(G13D), which stimulates YB-1 phosphorylation, we hypothesized that complete inhibition of P-YB-1 in HCT116 cells as a result of a higher concentration of LJI308 might be due to the higher level of YB-1 phosphorylation in these cells compared to that in SW48 cells. The data presented in Figure 3B support this conclusion and indicate that the P-YB-1 level in HCT116 cells was approximately 2.5 times higher than that in SW48 cells. Furthermore, using the CaCo2 CRC cell line, we showed that the conditional overexpression of oncogenic KRAS(G12V) led to an approximately three-fold greater stimulation of phospho-YB-1 (Figure 3C). Importantly, the difference in YB-1 phosphorylation level was not due to increased expression of total YB-1 (Figure S2). Together, these data indicated that KRAS(G12V) and KRAS(G13D) mutations stimulate YB-1 phosphorylation, which requires a higher concentration of RSK inhibitor for inhibition.

### 2.4. Upregulation of Akt after RSK Targeting Interferes with the Antiproliferative Effect of the LJI308 RSK Inhibitor 

**Figure 4:**
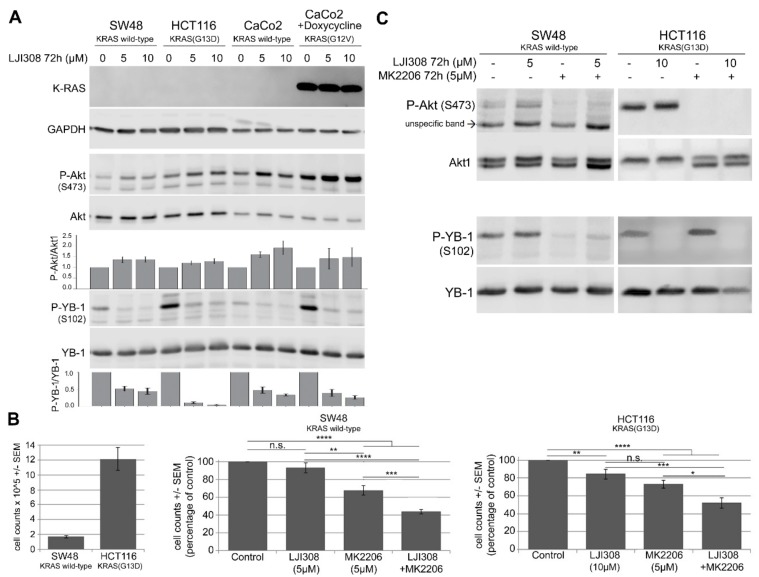
The upregulation of Akt after RSK targeting interferes with the antiproliferative effect of the LJI308 RSK inhibitor. (**A**) KRAS wild-type SW48 and CaCo2 cells as well as KRAS(G13D)-mutated HCT116 and doxycycline-treated CaCo2 cells were treated with the RSK inhibitor (5 µM and 10 µM) for 72 h. Phospho-Akt (S473), Akt1, phospho-YB-1 (S102), YB-1 and KRAS levels were detected by Western blotting. Glyceraldehyde 3-phosphate dehydrogenase (GAPDH) was detected as a loading control. The histogram represents the mean ratio of P/Akt/Akt1 and P-YB-1/YB-1 from at least four data: Two independent experiments normalized to one for the control (0 µM RSK inhibitor condition). A proliferation assay (**B**) and Western blotting (**C**) were performed in SW48 and HCT116 cells after treatment with the LJI308 RSK inhibitor and the MK2206 Akt inhibitor for 72 h. Statistical analyses indicated significant differences in cell proliferation after treatment with the indicated inhibitors (* *p* ≤ 0.05, ** *p* ≤ 0.01, *** *p* ≤ 0.001 and **** *p* ≤ 0.0001; n.s., nonsignificant). (**B**, left part) Comparison of absolute cell counts of control conditions in SW48 and HCT116 cells.

A functional crosstalk exists between the MAPK and PI3K pathways [35], in which the inhibition of one pathway leads to a compensatory activation of the other pathway [36,37]. Because inhibition of RSK downstream of ERK1/2 blocked YB-1 phosphorylation, we investigated if inhibition of RSK as a component of the MAPK pathway leads to the activation of Akt as a readout of PI3K activity. Seventy-two hours posttreatment with the LJI308 RSK inhibitor led to a dose-dependent inhibition of YB-1 phosphorylation in KRAS wild-type SW48 and CaCo2 cells as well as in KRAS-mutated HCT116 and CaCo2 cells after conditional expression of mutated KRAS (Figure 4A). Surprisingly, the inhibition of YB-1 phosphorylation by LJI308 was associated with the activation of Akt phosphorylation at S473. In line with the role of KRAS in YB-1 phosphorylation, YB-1 basal phosphorylation in HCT116 cells with endogenous mutated KRAS(G13D) and in CaCo2 cells with conditional expression of mutated KRAS(G12V) was markedly higher in KRAS wild-type SW48 and CaCo2 cells. In all cell lines tested in the stationary growth phase, the inhibition of YB-1 phosphorylation was associated with the activation of Akt. The level of Akt phosphorylation in CaCo2 cells after conditional expression of mutated KRAS was markedly higher than the phosphorylation in the parental cells. The low expression level of endogenous KRAS in *KRAS* wild-type SW48 and CaCo2 cells as well as in KRAS-mutated HCT116 cells is shown in Figure S3.

Assuming that the compensatory activation of Akt promotes the cell proliferation and survival that leads to the limited antiproliferative effect of the LJI308 RSK inhibitor, we performed a proliferation assay to test the effects of the inhibition of Akt and RSK alone and in combination in SW48 and HCT116 cells 72 h posttreatment. Compared to the MK2206 Akt inhibitor (5 µM), the LJI308 RSK inhibitor (5 µM) had a minor effect on cell proliferation in both cell lines tested (Figure 4B). However, simultaneous targeting of Akt and RSK significantly blocked cell proliferation. Under the proliferation treatment conditions, the reactivation of Akt after inhibition of YB-1 by the LJI308 RSK inhibitor (5 µM, 72 h) was blocked by the MK2206 Akt inhibitor (5 µM, 72 h) (Figure 4C). The level of YB-1 phosphorylation at S102 was not affected by MK2206. The combination of cetuximab with RAS/Akt inhibitors did not improve the response beyond the effect observed by the RAS/Akt inhibitors alone (Figure S4). This result was expected because cetuximab does not stimulate YB-1 activation, which can be inhibited by the LJ308 RSK inhibitor.

### 2.5. Dual Targeting of the RSK/YB-1 Pathway and Akt Enhances the Antiproliferative Effect of 5-FU in KRAS(G13D)-Mutated HCT116 Cells but Not in KRAS Wild-Type SW48 Cells by Stimulating Apoptosis 

**Figure 5:**
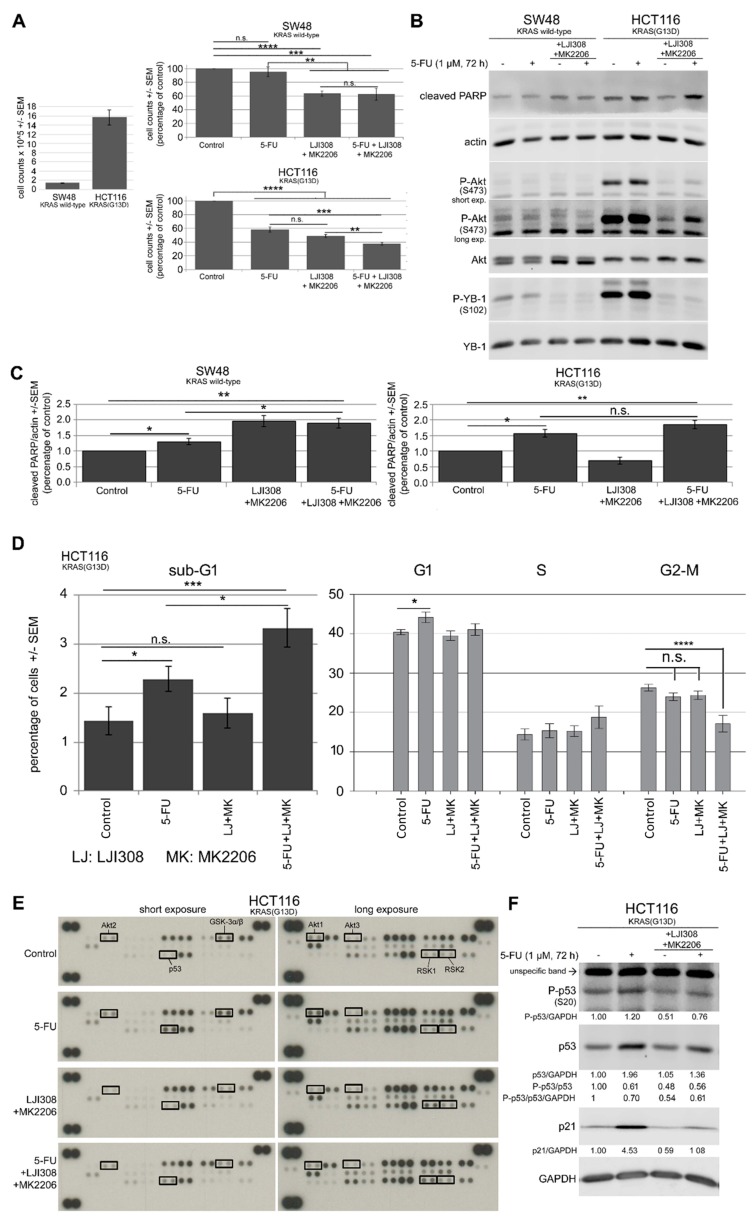
Dual targeting of the RSK/YB-1 pathway and Akt enhances the antiproliferative effect of 5-FU in KRAS(G13D)-mutated HCT116 cells but not in KRAS wild-type SW48 cells by stimulating apoptosis. KRAS wild-type SW48 and KRAS(G13D)-mutated HCT116 cells were treated with 5-FU (1 µM), a combination of the RSK and Akt inhibitors (5 µM LJI308 for SW48 and 10 µM LJI308 for HCT116 cells; 5 µM MK2206) or a combination of the inhibitors with 5-FU for 72 h. (**A**) A proliferation assay was performed as described in the Materials and Methods section, and the mean value of 9 data points from three independent experiments is shown. (**B**) After the indicated treatments, protein samples were isolated, and the levels of P-YB-1, P-Akt, YB-1, Akt1 and cleaved PARP were determined by Western blotting. Actin was detected as a loading control. (**C**) The ratio of cleaved PARP to actin was evaluated by densitometry from three independent experiments and graphed. (**D**) The percentage of sub-G1, G1, S, and G2/M cells after the described treatment was investigated by fluorescence-activated cell sorting (FACS) analysis, as described in the Materials and Methods section. Data represent the mean value of 10 data points from four independent experiments for sub-G1 cells and 9 data points from three independent experiments for G1, S and G2-M phases. Asterisks indicate significant differences between the indicated groups (* *p* ≤ 0.05, ** *p* ≤ 0.01, *** *p* ≤ 0.001 and **** *p* ≤ 0.0001). (**E**) A phospho-mitogen-activated protein kinase (MAPK) array was performed in HCT116 cells according to the manufacturer’s protocol after the described treatments. (**F**) Western blot analysis was performed to analyze P-p53 (S20), p53, p21 and GAPDH in the protein samples, which were applied to the phospho-MAPK array. Densitometry values below each blot represent the ratio of the indicated protein to GAPDH normalized to 1 under control conditions. (**A**, left part). Comparison of absolute cell counts of control condition in SW48 and HCT116 cells. n.s.: nonsignificant.

Treatment with 5-FU led to the activation of YB-1 by phosphorylation at S102 in HCT116 KRAS(G13D)-mutated cells (Figure 2A). Likewise, targeting RSK blocked YB-1 phosphorylation. However, RSK inhibition led to the activation of Akt in all the cell lines tested. Because HCT116 cells presented both YB-1 phosphorylation after treatment with 5-FU and Akt phosphorylation after inhibition of YB-1 by RSK inhibition, we sought to determine if dual targeting of the RSK/YB-1 pathway and Akt enhances the antiproliferative effect of 5-FU. Figure 5A indicates that 5-FU (1 µM, 72 h) markedly inhibited the proliferation of HCT116 cells but not SW48 cells. The combination of the RSK and Akt inhibitors significantly inhibited cell proliferation in both cell lines tested. Interestingly, the dual targeting of RSK and Akt for 72 h did not affect the response of SW48 cells to 5-FU, but it significantly enhanced the effect of 5-FU in HCT116 cells (Figure 5A).

In parallel to the proliferation assay, the status of phospho-YB-1 and phospho-Akt as well as poly (ADP-ribose) polymerase (PARP) cleavage was analyzed as an indication of apoptosis in both cell lines (Figure 5B). The LJI308 RSK inhibitor and the MK2206 Akt inhibitor blocked the phosphorylation of YB-1 and Akt, respectively. In line with the data shown in Figure 2, 5-FU induced YB-1 and Akt phosphorylation in HCT116 cells but not in SW48 cells. Furthermore, 5-FU induced PARP cleavage in both cell lines, whereas the dual targeting of RSK and Akt induced PARP cleavage in SW48 cells but not in HCT116 cells (Figure 5B,C). The triple combination of the inhibitors of RSK and Akt with 5-FU stimulated PARP cleavage only in HCT116 cells but not in SW48 cells.

To further investigate PARP cleavage-mediated apoptosis, a cell cycle analysis of HCT116 cells was performed. The results shown in Figure 5D indicate that single treatment with 5-FU caused a significant increase in apoptotic cells, as shown by the enhanced percentage of cells in the sub-G1-phase. Consistent with stimulated PARP cleavage after the triple combination treatment with 5-FU and the inhibitors, the triple combination significantly increased the percentage of apoptotic cells compared to the effect of 5-FU alone. The combination of the two inhibitors did not induce apoptosis (Figure 5D).

To investigate the underlying signaling pathway involved in apoptosis, a human phospho-MAPK array was utilized to analyze the expression and activation of major proteins/kinases directly or indirectly regulated in this pathway by modulating YB-1 phosphorylation downstream of the MAP kinase in HCT116 cells. Figure 5E indicates that the phosphorylation of Akt1 and Akt2 was increased after treatment with 5-FU in association with stimulated phosphorylation of the Akt substrate GSK-3α/β, which reflects Akt kinase activity. Treatment with MK2206 fully blocked GSK-3α/β phosphorylation and, thus, Akt activity. Surprisingly, RSK isoforms 1 and 2 showed stronger phosphorylation after treatment with the LJI308 RSK inhibitor. Additionally, data from this assay revealed that 5-FU induced p53 phosphorylation at S46, which was inhibited by the combination of the Akt and RSK inhibitors. Because p53 regulates apoptosis and cell cycle arrest by transactivation of p21 [38,39], the p53 expression level, p53 phosphorylation level at S20 and p21 target gene expression level were confirmed by Western blotting. Figure 5F shows that 5-FU stimulated expression of p53 in association with the enhanced phosphorylation at S46 (Figure 5E) and S20 (Figure 5F) as well as the expression of p21. Because the ratios of P-p53/p53 and P-p53/p53/ glyceraldehyde 3-phosphate dehydrogenase (GAPDH) were not enhanced in the 5-FU condition, p53 phosphorylation was enhanced at both sites as a consequence of enhanced protein expression. p53 and p21 expression led to accumulation of cells in G1 arrest (Figure 5D). Treatment with the combination of Akt and RSK inhibitors blocked 5-FU-induced p53 and p21 expression, which released cells from G1 arrest, and cells released from G1 phase did not undergo synthesis and accumulated in S phase (Figure 5D). 

### 2.6. Dual Targeting of the RSK/YB-1 Pathway and Akt Enhances the Clonogenic Inactivation by 5-FU in KRAS(G13D)-Mutated HCT116 Cells 

**Figure 6:**
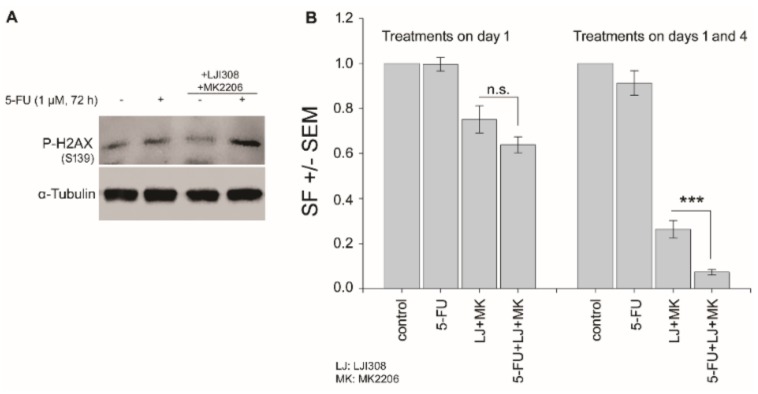
Dual targeting of the RSK/YB-1 pathway and Akt enhances the clonogenic inactivation by 5-FU in KRAS(G13D)-mutated HCT116 cells. Cells were treated with 5-FU (1 µM), RSK/Akt inhibitors (10 µM of LJI308 and 5 µM of MK2206) or a combination of the inhibitors with 5-FU for 72 h. (**A**) Level of P-H2AX (S139) was detected by Western blotting. α-Tubulin was detected as loading control. (**B**) Clonogenic assay was performed as described in the Materials and Methods section. Cells were plated in 6-well plates (250 cells/well) and were treated as indicated either on day 1 (right part of the histogram) or on days 1 and 4 (right part of the histogram). 9 days later, colonies that formed were stained and counted. Survival fractions were calculated as described in the Materials and Methods section. Data represent the mean survival fraction (SF) ± SEM from three biologically independent experiments (18 data) for treatments on day 1 and two biologically independent experiments (12 data) for treatments on days 1 and 4. Asterisks indicated significant inhibition of clonogenic activity by the combination of RSK/Akt inhibitors with 5-FU compared to the effect of RSK/Akt inhibitors alone (*** *p* ≤ 0.001). n.s.: nonsignificant.

5-FU induces DNA damage, and both YB-1 and Akt play roles in various DNA damage repairs. Thus, it is expected that the proposed triple combination therapy would interfere with the repair of DNA damage, thereby leading to cell death through mitotic catastrophe, which is not reflected by apoptosis. The status of phosphorylation of H2AX as a powerful tool for detecting genotoxicity supports this hypothesis. As shown in Figure 6A, the level of phospho-H2AX was enhanced in the combination of 5-FU with RSK/Akt inhibitors. Interference with DNA repair leads to clonogenic inactivity that can be validated by clonogenic assay. To this aim, we tested the clonogenic activity of cells after treatment with 5-FU and RSK/Akt inhibitors alone or in combination. Following plating, cells were treated either once on day 1 or twice on days 1 and 4. In KRAS(G13D)-mutated HCT116, single treatment with 5-FU (1 µM) on day 1 after plating did not affect clonogenic activity (Figure 6B). A slight but not significant effect was observed when cells were treated with 5-FU on days 1 and 4. RSK/Akt inhibitors alone significantly inhibited clonogenicity in cells treated either on day 1 or on day 1 and 4. Interestingly, combination of 5-FU with RSK/Akt inhibitors inhibited clonogenic activity in both treatment schedules. However, when compared with the effect of RSK/Akt inhibitors, a significant inhibition of clonogenic activity was observed after combination with 5-FU in cells, which were treated twice on days 1 and 4 (Figure 6B). A similar experiment was performed in KRAS wild-type SW48 cells. However, the plating efficiency (number of colonies with more than 50 cells/number of cells seeded) of these cells in control-treated condition was extremely low, and further analysis was not possible.

**Figure 7:**
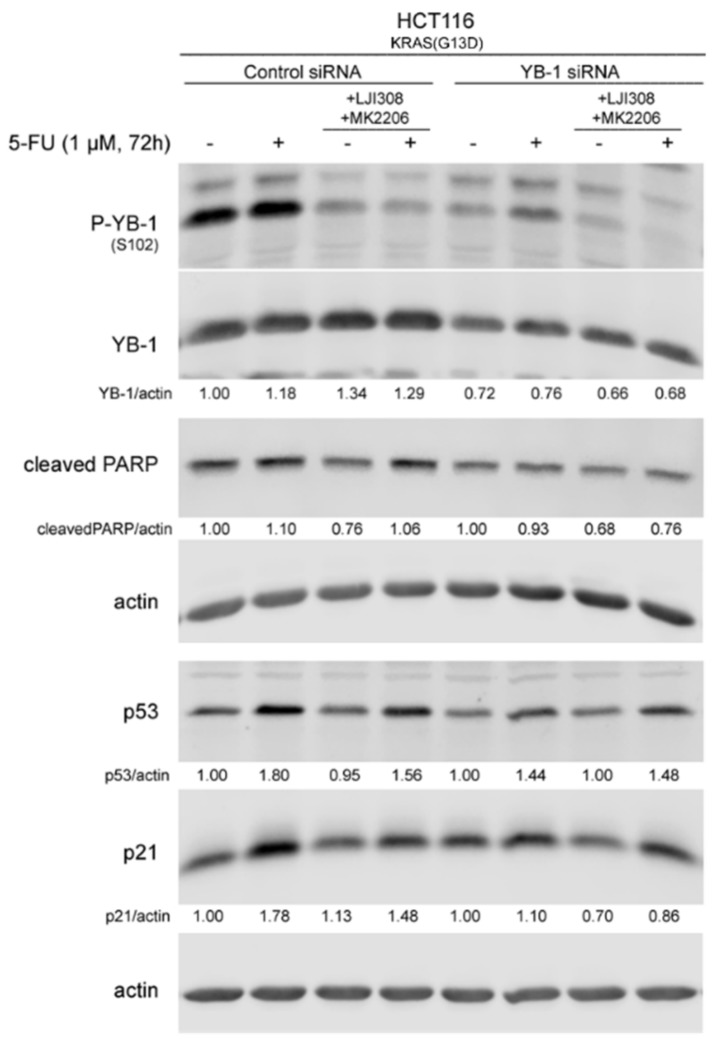
The 5-FU-induced expression of p53 and p21 as well as the inhibitory effect of RSK and Akt inhibitors in KRAS(G13D)-mutated HCT116 cells are YB-1 dependent. KRAS(G13D)-mutated HCT116 cells were transfected with either 50 nM control siRNA or YB-1 siRNA. Twenty-four hours after transfection, cells were treated with and without 5-FU, LJI308 and MK2206 for 72 h, and protein samples were isolated. Phosphorylation and expression of the indicated proteins were analyzed by Western blotting. All densitometry values were obtained from the ratio of the indicated proteins to actin. The values for YB-1 were normalized to nontreated siRNA-transfected conditions. The ratio of YB-1 to actin was normalized to the nontreated control siRNA-transfected cells.

Because 5-FU induced YB-1 activation, p53 expression and p21 expression, which were blocked by the combination of the RSK and Akt inhibitors, we next determined if these effects are dependent on YB-1 by knocking down YB-1 using siRNA (Figure 5F). Similar to the data shown in Figure 5B,C, 5-FU-induced PARP cleavage was stimulated by the combination of the RSK and Akt inhibitors (Figure 7), but this effect was not observed after YB-1 knockdown. Furthermore, YB-1 knockdown led to decreased expression of p53 and 5-FU-induced p53 expression after YB-1 knockdown was not further affected by additional treatment with inhibitors (Figure 7). Thus, these data indicated that YB-1 plays a key role in the expression of p53 and in the regulation of apoptosis pathways.

### 2.7. YB-1 Is Highly Phosphorylated in CRC Patient Tumor Tissues

**Figure 8:**
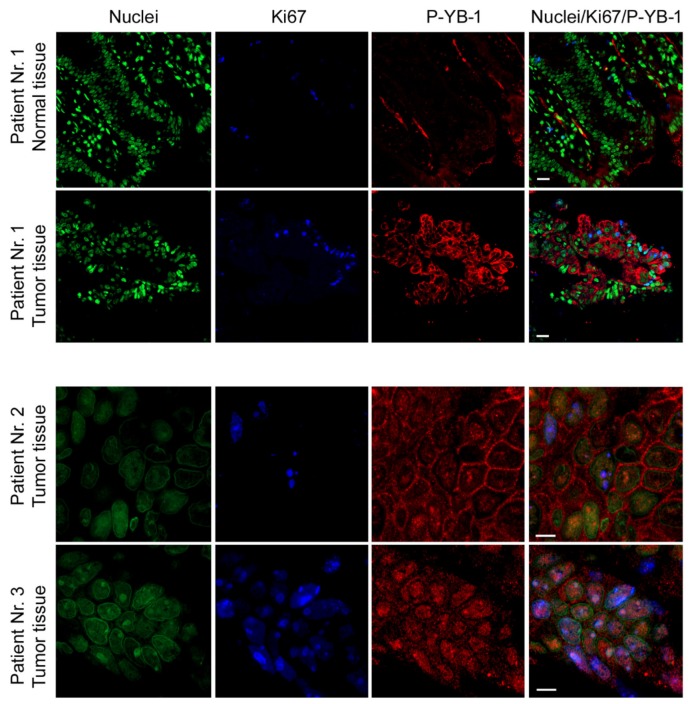
YB-1 is highly phosphorylated in CRC patient tumor tissues. Paraffin-embedded sections were used for immunofluorescence staining of phospho-YB-1 in tumor tissues and the normal tissue obtained from patient no. 1. Nuclei were stained with Yopro. Ki67 was used as an indicator of cell proliferation. The sections were analyzed with a confocal laser scanning microscope. Scale bars represent 20 µm for images from patient no. 1 and 0.5 µm for patients no. 2 and no. 3.

Because the in vitro data showed that YB-1 was highly phosphorylated in the different CRC cell lines tested and that the phosphorylation was stimulated by point mutations in KRAS, we tested the phosphorylation status of YB-1 in tumor tissues from three CRC patients. Samples from patients undergoing surgery, excluding samples from patients with neoadjuvant chemo-/radiotherapy, were used. Data from immunofluorescence staining revealed that YB-1 was highly phosphorylated in the tumor samples obtained from the three CRC patients. The level of phospho-YB-1 in the normal tissue was markedly lower than that in the corresponding tumor tissue obtained from patient no. 1 (Figure 8). Interestingly, YB-1 phosphorylation in tumor samples from patient no. 1 was localized mainly in the cytoplasm, while it was observed in both subcellular fractions in patient no. 2 (Figure 8). In patient no. 3, YB-1 phosphorylation was mainly localized in the nucleus.

## 3. Discussion

Despite various therapeutic approaches, colorectal cancer (CRC) is still one of the most lethal sex-independent cancers worldwide. 5-FU-based combined chemotherapy is the first-line treatment for CRC. Furthermore, EGFR targeting is also a treatment approach for CRC that is applicable only for patients with KRAS wild-type but not for patients with a KRAS mutation. The KRAS mutational-based limitation of the latter approach and overcoming primary or acquired chemoresistance are challenges in CRC treatment. For the first time, the present study demonstrated that CRC cells expressing mutations in KRAS stimulated phosphorylation of the multifunctional Y-box binding protein-1 (YB-1) at S102 via activation of p90 ribosomal S6 kinase (RSK). Long-term treatment with an RSK inhibitor unexpectedly led to the reactivation of Akt, which diminished the anti-proliferative effect of the RSK inhibitor independent of KRAS mutational status. Thus, dual targeting of RSK and Akt may be an efficient strategy to block the proliferation of both KRAS wild-type and KRAS-mutated CRC cells. Furthermore, this molecular targeting approach also sensitized KRAS-mutated CRC cells to 5-FU.

CRC cancer cells, which differ in their genetic features, responded differently to anticancer treatments. In line with previous reports [4,5,6], the response to cetuximab depended on KRAS status. Cetuximab inhibited cell proliferation in KRAS wild-type cells, while the proliferation of cells with endogenous expression of KRAS(G12V) was moderately inhibited. Cetuximab treatment did not affect proliferation of cells with endogenous mutated KRAS(G13D). The present data agreed with the report by Napolitano et al. showing that SW48 cells are sensitive to cetuximab and that HCT116 cells are resistant to cetuximab [40]. Napolitano et al. [40] also reported that cetuximab has a dose-dependent antiproliferative effect in SW480 cells, although the effect is much weaker than the effect in SW48 cells. A lack of antiproliferative effect of cetuximab was also observed after conditional overexpression of KRAS(G12V). The differential effect of endogenous mutated KRAS in SW480 cells versus conditional expression of mutated KRAS in CaCo2 cells indicated that additional parameters of KRAS mutation in cells may influence the response of KRAS-mutated cells to cetuximab. Cellular functions are differentially affected by various point mutations in the KRAS gene. For instance, stimulation of metastasis in the CRC model is much stronger after KRAS(G12V) mutation than after KRAS(G13D) mutation [41]. The present study showed that the impacts of different mutations in KRAS (i.e., KRAS(G12V) and KRAS(G13D)) in response to cetuximab and YB-1 phosphorylation stimulation were similar. Both mutations stimulated YB-1 phosphorylation and diminished the response to cetuximab. Interestingly, in the present study, the expression of KRAS(G12V) significantly reduced the proliferation of CaCo2 cells. Because doxycycline did not affect cell proliferation in either KRAS wild-type or KRAS-mutated CRC cells (see Figure S1), the reduced proliferation after overexpression of mutated KRAS may be due to the hyperactivation of the MEK-ERK-cyclin D pathway stimulating senescence by producing p15, p16 and p19 proteins [42]. According to a recent report by Chu et al. [43], mutant KRAS promotes liver metastasis of colorectal cancer by stimulating YB-1-dependent IGF-1 receptor expression. The present study showed that the enhanced phosphorylation of YB-1 was not a consequence of stimulated protein expression (Figure 2A, Figure 3A and Figure 4A,B). In fact, the YB-1 protein expression level in KRAS wild-type SW48 cells normalized to actin was significantly higher than the expression level in KRAS(G13D)-mutated HCT116 cells (Figure S2). Furthermore, in KRAS wild-type CaCo2 cells, the conditional expression of KRAS(G12V) did not affect the expression of YB-1 (Figure S2). Further studies are necessary to clarify these conflicting observations.

The present study showed that KRAS-mutated cells had significantly higher phospho-YB-1 than KRAS wild-type cells, which agreed with the role of KRAS in YB-1 phosphorylation in breast cancer cells [30]. Targeting RSK blocks the proliferation of breast cancer cells via the inhibition of YB-1 [31,32,44]. The present study showed that complete inhibition of YB-1 phosphorylation by a RSK inhibitor had only a marginal effect on cell proliferation. Similarly, Aronchik et al. reported that the inhibition of RSK fully suppresses YB-1 phosphorylation but does not affect cell growth in an attached setting [31]. Together, these data indicate that RSK inhibition along with the inhibition of YB-1 phosphorylation stimulates the activation of a pathway that stimulates cell proliferation and survival. The PI3K/Akt pathway is one of the most important survival pathways that is deregulated in different cancers, and it stimulates DNA damage repair [45,46]. Among the different pathways tested, RSK inhibition led to the activation of Akt independent of KRAS mutational status. The reactivation of Akt in the present study was supported by a functional interaction between RSK and Akt, as described by Moritz et al. [47], as well as the crosstalk between the MAPK/ERK/RSK and PI3K/Akt pathways [36,37]. Akt regulates many cellular functions, including growth, proliferation, and survival. Thus, reactivation of Akt by RSK inhibition diminished the antiproliferative effect of the LJI308 RSK inhibitor. As a result, the combination of Akt and RSK inhibitors led to enhanced antiproliferative effects compared to each treatment alone as shown in Figure 4. These data agreed with the report by Kotake et al., thus supporting the role of YB-1 in stimulating cell cycle progression [48].

5-FU-based combined chemotherapy is the first-line treatment for CRC. The antitumor activity of 5-FU is mainly due to the inhibition of the thymidylate synthase enzyme, resulting in imbalances in the deoxynucleotide pool, thereby blocking DNA synthesis [49]. Moreover, 5-FU metabolites are incorporated into DNA, leading to DNA damage [49]. The base excision repair (BER) and mismatch repair (MMR) pathways are the most relevant pathways in response to 5-FU-induced DNA damage [50]. YB-1 is involved in DNA repair, e.g., BER, MMR and double-strand break repair, via interactions with the DNA repair machinery [15]. Thus far, the interaction of YB-1 with the Ku80, MSH2, PCNA, PARP1 and PARP2 repair proteins has been described [16,17,51]. According to the role of YB-1 in DNA repair, 5-FU-induced YB-1 activity can protect cells against 5-FU. It is known that the activation of Akt leads to 5-FU resistance in colorectal cancer [52,53]. The present study showed that 5-FU induces Akt activation independent of KRAS mutational status but that 5-FU-induced stimulation of YB-1 only occurs in KRAS(G13D)-mutated cells. This effect was not observed when KRAS(G12V) was overexpressed. Thus, it can be concluded that the activation of YB-1 and Akt in KRAS(G13D)-mutated cells results in protection against 5-FU-induced cell death, e.g., apoptosis and mitotic catastrophe. This conclusion was supported by biochemical studies regarding the regulation of p53, p21 and PARP cleavage as well as the induction of apoptosis after treatment with 5-FU in combination with the RSK and Akt inhibitors (Figure 5B–D). YB-1-dependent expression of p53 and p21 after 5-FU treatment agreed with a previous report indicating activation of the YB-1/p53 pathway, leading to glioblastoma resistance to temozolomide [54]. Combination of the RSK and Akt inhibitors with 5-FU enhanced 5-FU-induced apoptosis by approximately 35%, which may be further enhanced after multiple treatments, which is commonly applied in clinical settings. Akt stimulates DNA damage repair [15,45,46]. Thus, the combination of RSK and Akt inhibitors with 5-FU may also interfere with repair of 5-FU-induced DNA damage. The phosphorylation of histone H2AX at Ser-139 was enhanced after treatment with the RSK/Akt inhibitors and 5-FU, indicating residual DNA damages. Unrepaired DNA damage leads to cell death due to mitotic catastrophe, which may not be reflected by apoptosis. In fact, the clonogenic assay data support this conclusion. Based on the data obtained from this assay, we propose that the advantage of triple combination of the RSK/Akt inhibitors with 5-FU in cellular clonogenic inactivation can be observed when cells carry a certain level of DNA damage. This threshold of DNA damage, which is necessary for clonogenic inactivation of KRAS(G13D)-mutated HCT116 cells, seems to be induced by twice treatment with 1 µM of 5-FU. 

Nuclear YB-1 expression status correlates with poor prognosis of patients with stage III CRC [20] and estrogen receptor-negative prostate cancer [55]. In the present study, YB-1 phosphorylation staining of CRC patient samples revealed that YB-1 was highly phosphorylated in tumor tissues and that phospho-YB-1 was expressed in the nucleus in two out of three samples. Interestingly, the YB-1 phosphorylation level in normal tissue, which was only available from one patient, was low. These data indicated that YB-1 is most likely a tumor-specific marker. Based on the biochemical data in the present study, YB-1 maybe be targeted for treatment of colorectal cancers in combination with chemotherapy.

## 4. Materials and Methods 

### 4.1. Cell Lines

The colorectal cancer cell lines KRAS wild-type SW48 (ATCC, CCL-231), KRAS(G12V)-mutated SW480 (ATCC, CCL-228) and KRAS(G13D)-mutated HCT116 (ATCC, CCL-247) were used. Additionally, we used the colorectal carcinoma cell line CaCo2 that was stably transfected with doxycycline-inducible KRAS(G12V) [33]. The cells were cultured in Dulbecco’s modified Eagle’s medium (DMEM) (SW48, SW480) and in RPMI (HCT116) supplemented with 10% fetal calf serum (FCS) and 1% penicillin-streptomycin (PS). CaCo2 cells were cultured in low glucose DMEM + 10% FCS + 1% PS + 1% glutamine that was additionally supplemented with 5 µg/mL puromycin and 5 µg/mL blasticidin.

### 4.2. Antibodies and Reagents

The antibodies for Western blot analyses of P-YB-1 (S102) (#2900), YB-1 (#4202), P-Akt (S473) (#4060), GAPDH (#2118S), cleaved PARP (#9541S), P-p53 (S20) (#9287P), p53 (#9282S) and p21 (#2947S) were purchased from Cell Signaling Technology (Frankfurt, Germany). The Akt antibody (#610877) was purchased from BD Biosciences (Heidelberg, Germany). The KRAS antibody (#ab157255) and the antibody for immunofluorescence staining of P-YB-1 (#ab47162) were purchased from Abcam (Cambridge, UK). The actin antibody (#A2066) was purchased from Sigma-Aldrich (Taufkirchen, Germany). The anti α-tubulin antibody (cat. #CP06) was purchased from Calbiochem (Schwalbach, Germany). Anti-Ki67 (#A2066) was purchased from Agilent (Waldbronn, Germany). The Human Phospho-MAPK array kit was purchased from R&D Systems, Inc. (Minneapolis, MN, USA). Anti-phospho-Histone H2AX (Ser139) antibody (#05-636) was purchased from Merck Millipore (Darmstadt, Germany). The RSK inhibitor LJI308 (#S7871) and Akt inhibitor MK2206 (#S1078) were purchased from Selleckchem (Munich, Germany). 5-fluorouracil (#84300-VO) was purchased from Medac (Hamburg, Germany). Cetuximab was provided by the pharmacy at the University of Tuebingen (Tuebingen, Germany). Doxycycline (#A2951) was purchased from AppliChem (Darmstadt, Germany).

### 4.3. Western Blotting

Cells were treated according to each experiment, and protein samples were isolated as described previously [56]. Protein samples were subjected to sodium dodecyl sulfate-polyacrylamide gel electrophoresis (SDS-PAGE) and transferred to a nitrocellulose membrane by semidry blotting. The membranes were incubated with specific primary antibodies overnight at 4 °C followed by incubation with secondary antibodies for 1 h at room temperature. Proteins were visualized by an enhanced chemiluminescence detection system using the LI-COR Biosciences system (Bad Homburg, Germany). Whole blot can be found it Supplementary 2.

### 4.4. Proliferation Assay

Cells were seeded in 60 mm culture dishes and treated after 24 h. Cells were trypsinized and counted using a hemocytometer 72 h after treatment.

### 4.5. Cell Cycle Analysis

Cells were seeded in 100 mm culture dishes and treated accordingly after 24 h. Subsequently, 72 h after treatment, the cells were trypsinized, and floating cells were collected. Following centrifugation, cells were washed with PBS and fixed in chilled 70% ethanol. To perform flow cytometry analysis, cells were washed again with PBS and incubated with staining buffer (PBS + RNase A (10 µg/mL) + Propidium Iodide (PI) (50 µg/mL)) for 1 h. Cells were centrifuged and resuspended in FACS buffer (PBS + 1 mM EDTA + 5% FCS). The cell cycle distribution was evaluated by flow cytometry.

### 4.6. Human Phospho-MAPK Array

The phospho-MAPK array was performed according to the manufacturer’s instructions. Briefly, four nitrocellulose membranes spotted in duplicate with control and capture antibodies were blocked with blocking buffer for 1 h. After appropriate treatment, the cells were washed with ice-cold PBS and lysed with lysis buffer. The membranes were incubated with cell lysates overnight, followed by washing. Thereafter, membranes were incubated with horseradish peroxidase for 30 minutes, washed again, incubated with chemiluminescence reaction reagents for 1 minute and exposed to X-ray film.

### 4.7. Clonogenic Assay

Clonogenic survival of cells following indicated treatments was analyzed by means of colony formation assay as described before [57]. Cells were plated in 6-well plates and, after 24 h were treated as described in Results. Cultures were incubated for 9 days to allow for colony growth. Colonies of >50 cells were scored as survivors. Clonogenic fraction of treated cells wan normalized to the plating efficiency of vehicle-treated controls. Plating efficiency is described as number of colonies with more than 50 cells to the number of cells seeded.

### 4.8. Transfection with siRNA

Cells were seeded at a density of 150,000 cells/well in 6-well plates 24 h before transfection. Cells were transfected with 50 nM of either control siRNA or YB-1 siRNA. Twenty-four hours after transfection, the cells were treated with 5-FU (1 µM), LJI308 (10 µM) and MK2206 (5 µM) for 72 h according to the experiment.

### 4.9. Immunofluorescence Analysis

CRC patients undergoing surgery as a primary treatment were included in the study. Previous neo-adjuvant treatment or chemoradiotherapy were the exclusion criteria. The study was approved by the Ethics Committee of the Medical Faculty of the University of Tuebingen (confirmation #426/2013BO1). All patients provided signed, informed consent. Paraffin sections were deparaffinized and rehydrated. Slides were incubated in citrate buffer pH 6.0 (Thermo Scientific, Karlsruhe, Germany) using a pressure cooker. For immunofluorescence analysis, sections were blocked with donkey serum followed by incubation with rabbit antiserum to phospho-YB-1 and mouse antiserum to Ki67. The bound antibody was visualized by incubation with Cy3-donkey anti-rabbit serum and Cy5-donkey anti-mouse serum (Dianova, Hamburg, Germany). Nuclei were stained with Yopro (1:2000 Invitrogen, Karlsruhe, Germany). Sections were analyzed using a Zeiss LSM 800 confocal laser scanning microscope, with Zeiss ZEN 2.3 (blue edition) Software.

### 4.10. Statistical Analysis

Student’s *t*-test was performed to analyze statistically significant differences after the indicated treatments using SigmaPlot software (Systat Software Inc., Version 7.0, Erkrath, Germany). A *p*-value < 0.05 was determined to be significant.

## 5. Conclusions

In conclusion, due to the high level of phosphorylation of YB-1 in tumor tissues from CRC patients, cotargeting YB-1 and Akt is a suitable approach to reduce cell proliferation in CRC cells, independent of KRAS mutational status. Likewise, this approach potentiates 5-FU-induced cell death in KRAS(G13D)-mutated cells and can be a novel treatment strategy in cetuximab-resistant KRAS-mutated cells.

## Supplementary Materials

The following are available online at https://www.mdpi.com/2072-6694/11/4/562/s1, Figure S1: Doxycycline does not block proliferation of indicated CRC cell lines, Figure S2: KRAS mutation does not stimulate YB-1 expression in CRC cells, Figure S3: Level of endogenous KRAS in CRC cells. KRAS wild-type SW48 and CaCo2 cells as well as KRAS(G13D)-mutated HCT116 and doxycycline-treated CaCo2 cells were treated with an RSK inhibitor (5 µM and 10 µM) for 72 h, Figure S4: Combination of the RSK (LJ308, 20 µM) and Akt (MK2206, 5 µM) inhibitors with cetuximab (100 ng/mL) does not improve the effect of cetuximab as analyzed by the proliferation assay 72 h after treatment. Supplementary 2: Whole blot.
